# Prevention of Atherosclerosis Progression by 9-cis-****β****-Carotene Rich Alga *Dunaliella* in apoE-Deficient Mice

**DOI:** 10.1155/2013/169517

**Published:** 2013-09-23

**Authors:** Ayelet Harari, Revital Abecassis, Noa Relevi, Zohar Levi, Ami Ben-Amotz, Yehuda Kamari, Dror Harats, Aviv Shaish

**Affiliations:** ^1^The Bert W. Strassburger Lipid Center, Sheba Medical Center, Tel Hashomer, 5265601 Ramat-Gan, Israel; ^2^Sackler Faculty of Medicine, Tel-Aviv University, Ramat-Aviv, 69978 Tel-Aviv, Israel; ^3^N.B.T., Haarava Eilat, Israel

## Abstract

*Introduction*. **β**-Carotene-rich diet has been shown to be inversely associated with the risk of coronary heart disease. However, clinical trials using synthetic all-trans-**β**-carotene failed to demonstrate a beneficial effect. We therefore sought to study the effect of natural source of **β**-carotene, the alga *Dunaliella*, containing both all-trans and 9-cis-**β**-carotene on atherosclerosis. In a previous study we showed that 9-cis-**β**-carotene-rich powder of the alga *Dunaliella* inhibits early atherogenesis in low-density lipoprotein receptor knockout mice. *Aims*. The aims of the current work were to study whether diet enriched with *Dunaliella* powder would inhibit the progression of established atherosclerosis in old male apoE-deficient mice and to compare the effect of *Dunaliella* on lipid profile and atherosclerosis in a low-versus high-fat diet fed mice. *Methods*. In the first experiment, young mice (12 weeks old) were allocated into 3 groups: (1) low-fat diet; (2) low-fat diet + *Dunaliella* powder (8%); (3) low-fat diet + **β**-carotene-deficient *Dunaliella*. In the second experiment, old mice (7 months old) with established atherosclerotic lesions were allocated into 4 groups: (1) low-fat diet; (2) low-fat diet + *Dunaliella*; (3) high fat-diet; (4) high-fat diet + *Dunaliella*. *Results*. In young mice fed a low-fat diet, a trend toward lower atherosclerotic lesion area in the aortic sinus was found in the *Dunaliella* group compared with the control group. In old mice with established atherosclerotic lesion, *Dunaliella* inhibited significantly plasma cholesterol elevation and atherosclerosis progression in mice fed a high-fat diet. *Conclusion*. The results of this study suggest that a diet containing natural carotenoids, rich in 9-cis-**β**-carotene, has the potential to inhibit atherosclerosis progression, particularly in high-fat diet regime.

## 1. Introduction

*β*-Carotene is a main source of three major forms of vitamin A: retinol, retinal, and retinoic acid. High dietary consumption and plasma concentrations of *β*-Carotene have been shown to be inversely associated with the risk of coronary heart disease [1, 2]. Nevertheless, randomized clinical trials using synthetic all-trans-*β*-Carotene failed to demonstrate a beneficial effect on cardiovascular disease [3, 4]. Moreover, a meta-analyses of clinical trials with antioxidant supplementation includin synthetic *β*-Carotene led to the conclusion that *β*-Carotene may increase mortality. It was suggested that *β*-Carotene may act as a cocarcinogen, leading to the increased mortality [5, 6].

While the synthetic *β*-Carotene used in these trials comprised only the all-trans isomer, natural *β*-Carotene in human diet comprises several isomers, including all-trans and 9-cis-*β*-Carotene. The 9-cis isomer, which is found in several fruits and vegetables, is present in the highest levels in the halotolerant alga *Dunaliella* [7]. Under appropriate conditions, *β*-Carotene represents up to 10% of the dry weight of the alga. This *β*-Carotene is composed of approximately 50% all-trans and 50% 9-cis-*β*-Carotene isomers [8]. Due to these properties, we have used the algal powder as source for natural *β*-Carotene isomers.

*β*-Carotene is a precursor of vitamin A including its most biologically active form, retinoic acid. While all-trans-*β*-Carotene is a precursor off all-trans retinoic acid, the 9-cis isomer has been shown to be a precursor of 9-cis retinoic acid *in vitro* in human intestinal mucosa [9] and *in vivo* in a ferret perfused with 9-cis-*β*-Carotene [10] All-trans and 9-cis retinoic acid are ligands of nuclear receptors. While both are ligands of the nuclear receptor retinoic acid receptor (RAR), only 9-cis retinoic acid binds to retinoid X receptor (RXR). Retinoic acid and other *β*-Carotene metabolites are known to affect multiple metabolic pathways involved in the development of atherosclerosis [11]. We hypothesized that 9-cis-*β*-Carotene potentially acts via its downstream metabolites, including 9-cis retinoic acid. In a previous study we demonstrated that synthetic *β*-Carotene does not inhibit atherogenesis in the apoE^−/−^ mouse model of atherosclerosis [12]. Moreover, old apoE^−/−^ mice, supplemented with a combination of vitamin C, vitamin E, and *β*-Carotene, did not show beneficial effects on lesion progression or composition [13]. However, we recently showed that *β*-Carotene-rich powder of the alga *Dunaliella* inhibits plasma cholesterol elevation in Low-Density Lipoprotein LDL-Receptor-knockout (LDL-R^−/−^) mice fed a high-fat diet. Importantly, atherosclerotic lesion area in *Dunaliella*-treated mice was 60–83% lower compared to mice fed a high-fat diet alone. In addition, we showed that this effect is associated with dietary 9-cis-*β*-Carotene content [14]. In the LDL-R^−/−^ model, we studied the effect of *Dunaliella* on early atherogenesis; therefore, in the present study we aimed to investigate the effect of *Dunaliella* on the progression of established atherosclerosis, using apoE^−/−^ mouse model characterized with advanced atherosclerotic lesions. 

## 2. Materials and Methods

### 2.1. Animals and Diets

 ApoE^−/−^ mice (C57BL/6 background, Jackson Laboratories) were used in this study. Mice were housed in plastic cages on a 12 : 12 hour light/dark cycle with free access to water and diet. Mice were distributed evenly among the treatment groups according to their plasma cholesterol and triglyceride levels. Blood samples were taken after an overnight fasting at time zero and throughout the experiments. The Animal Care and Use Committee of Sheba Medical Center, Tel-Hashomer, approved all animal protocols. Two commercial diets were used: a nonpurified, low-fat diet (18% protein, 5% fat; TD2018, Harlan Teklad) and a semipurified high-fat diet (17.3% protein, 21.2% fat, 0.2% cholesterol; TD88137, Harlan Teklad). The feed was prepared as described before [14]. Feed was replaced every other day to minimize oxidation and degradation of the ingredients. The *Dunaliella* powder (Nikken Sohonsha Corporation, Gifu, Japan) contained 50% all-trans and 50% 9-cis-*β*-Carotene. The total *β*-Carotene content of the feed was 0.6%. 

### 2.2. Study Design

In the first experiment, forty five, 12-week-old male mice, fed with low-fat diet were allocated into 3 groups: (1) diet alone (control group); (2) diet enriched with 8% *Dunaliella bardawil* powder; (3) diet enriched with *β*-Carotene-deficient *Dunaliella* powder (8%). The treatment lasted 9 weeks. The *β*-Carotene deficient *Dunaliella* powder was prepared by exposing the original powder to air for 2 weeks, resulting in complete degradation of carotenoids.

 In the second experiment, we used 7-month-old male mice (*n* = 70). Ten mice were killed at time zero to evaluate aortic lesion before treatment. The other 60 mice were allocated into 4 treatment groups. The treatment lasted 8 weeks: (1) low-fat diet; (2) low-fat diet enriched with 8% *Dunaliella bardawil*; (3) low-fat diet for 2 weeks followed by a high-fat diet for 6 weeks; (4) low-fat diet enriched with 8% *Dunaliella bardawil* powder for 2 weeks followed by a high-fat diet enriched with 8% *Dunaliella bardawil* powder for 6 weeks. 

### 2.3. Laboratory Methods

Colorimetric enzymatic procedures were used to measure plasma total cholesterol (Chol, Roche/Hitachi, Roche Diagnostics) and triglycerides (Infinity, Thermo Electron Corporation). The samples were analyzed at the time of the collection. The Cobas Mira autoanalyzer (Roche) was used for lipid measurements. 

*β*-Carotene isomers levels in the feed and in the liver were determined by high-performance liquid chromatography according to Shaish et al. [12].

Quantification of atherosclerotic fatty streak lesions was done by calculating the lesion size in the aortic sinus [12]. The heart and upper section of the aorta were removed and embedded in Optimal Cutting Temperature (OCT) compound (Miles Inc.). Every other section (5–10 *μ*m thick) throughout the aortic sinus (400 *μ*m) was taken for analysis. The distal portion of the aortic sinus was recognized by the aortic valve, located between the left ventricular outflow tract and the ascending aorta. Sections were evaluated for fatty-streak lesions after staining with oil-red O. Lesion area analysis was performed by an examiner unfamiliar with the tested specimen using Imagepro software (Media Cybernetics, USA).

 Plasma lipoproteins were separated by size exclusion chromatography using a superose-6 column (1 × 30 cm) on a fast protein liquid chromatography (FPLC) system (Pharmacia), as described before [15]. Briefly, a 200 *μ*L aliquot of pooled plasma from each experimental group was injected into the column and separated with buffer containing 0.15 mmol/L NaCl, 0.01 mmol/L NaHPO_4_, and 0.1 mmol/L EDTA, pH 7.5, at a flow rate of 0.5 mL/min.

### 2.4. Statistical Analysis

 Analysis of variance (ANOVA) and Student's *t*-test were used to compare differences in the lesion area. Repeated measures analysis of variance was used to compare the differences in plasma cholesterol levels between the groups. Tukey's post hoc test was used to test all pairwise comparisons among means. *P* < 0.05 was accepted as statistically significant. Data were analyzed with SPSS 12 (SPSS Inc., USA). 

## 3. Results

### 3.1. Liver *β*-Carotene Content

**Figure 1:**
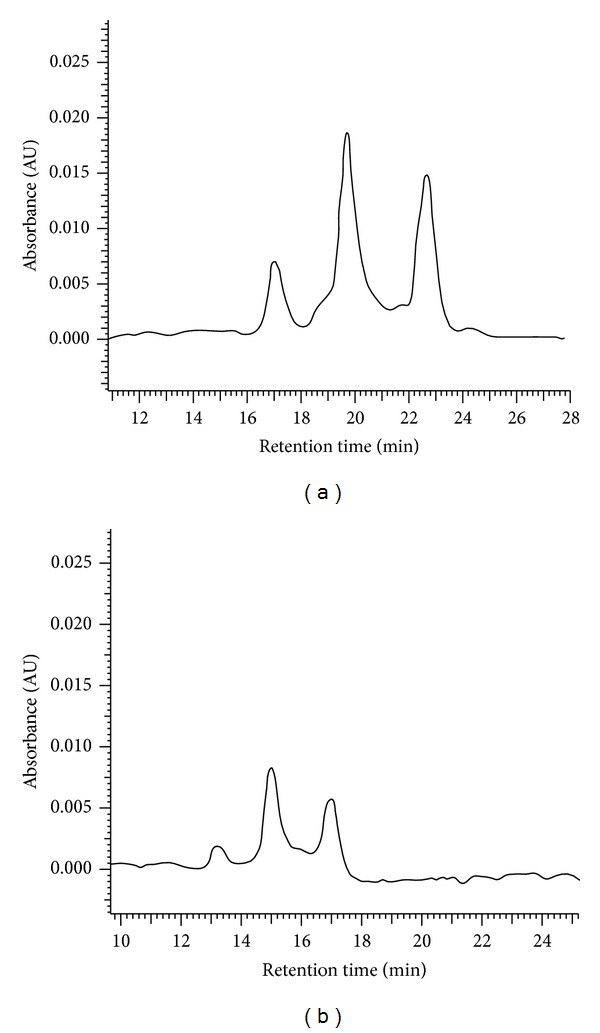
HPLC chromatograms of carotenoids in *Dunaliella* powder (a) and in the mouse liver (b). Carotenoids were separated on C18 HPLC column and detected by 450 nm absorbance.

First, we analyzed carotenoid levels in the mice feed and liver extracts from low-fat and high-fat-fed mice (Figure 1) to verify that *β*-Carotene from the algal powder accumulated in tissues following its absorption. As expected in mice, no *β*-Carotene was detected in plasma in all groups. In contrast, both all-trans and 9-cis-*β*-Carotene were detected in livers of *Dunaliella*-treated mice fed either a low- or high-fat diet, whereas no *β*-Carotene was found in livers of the control groups (data not shown). The ratio of 9-cis to all-trans-*β*-Carotene in liver extracts was 2 : 3 in the *Dunaliella*-fed groups, which was similar to the isomers ratio in the *Dunaliella* powder. These results show that the two isomers are absorbed and distributed to tissues, in a ratio similar to the ratio found in the mouse feed and that most of 9-cis-*β*-Carotene is not converted to all-trans-*β*-Carotene. 

### 3.2. Plasma Levels of Lipids and Lipoproteins

**Figure 2:**
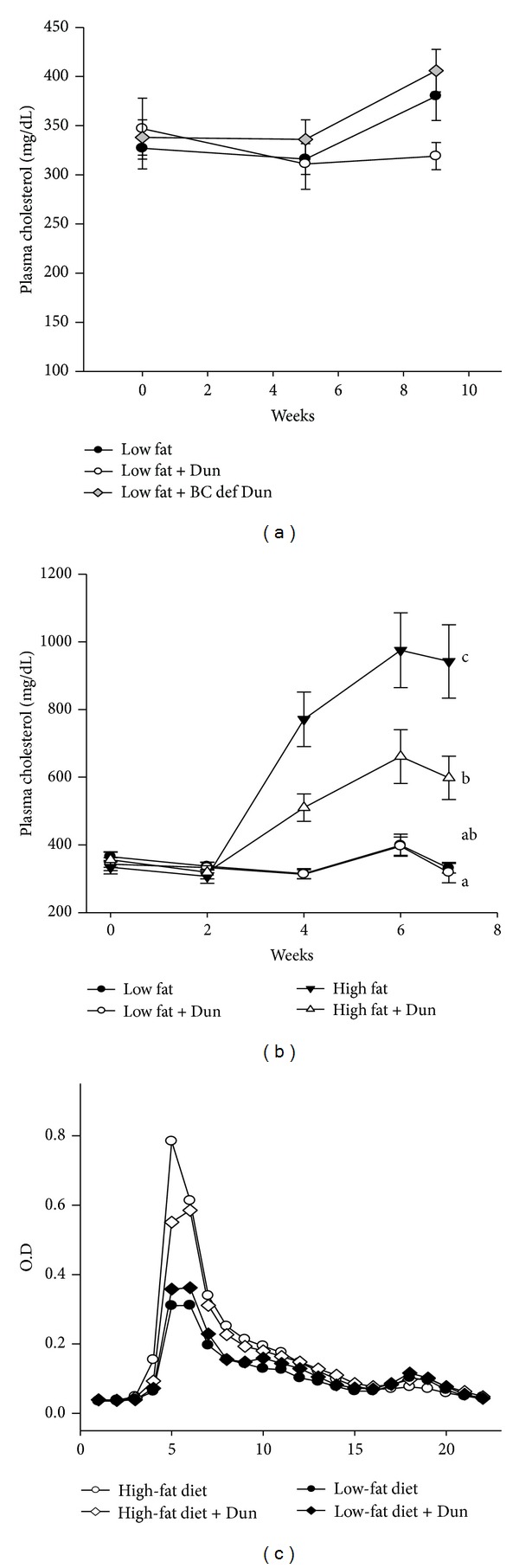
Plasma cholesterol levels in apoE^−/−^ mice fed (a) a low-fat diet, low-fat diet + *Dunaliella* (Dun), or low-fat diet + *β*-Carotene-deficient *Dunaliella* (BC def Dun) (first experiment); (b) low-fat diet, low-fat diet + Dun, high-fat diet or high-fat diet + Dun (second experiment). (c) FPLC chromatogram of the groups (second experiment). Values are means ± SE, *n* = 7–15 in each group. Means at a time without a common letter differ, *P* < 0.05.

**Figure 3:**
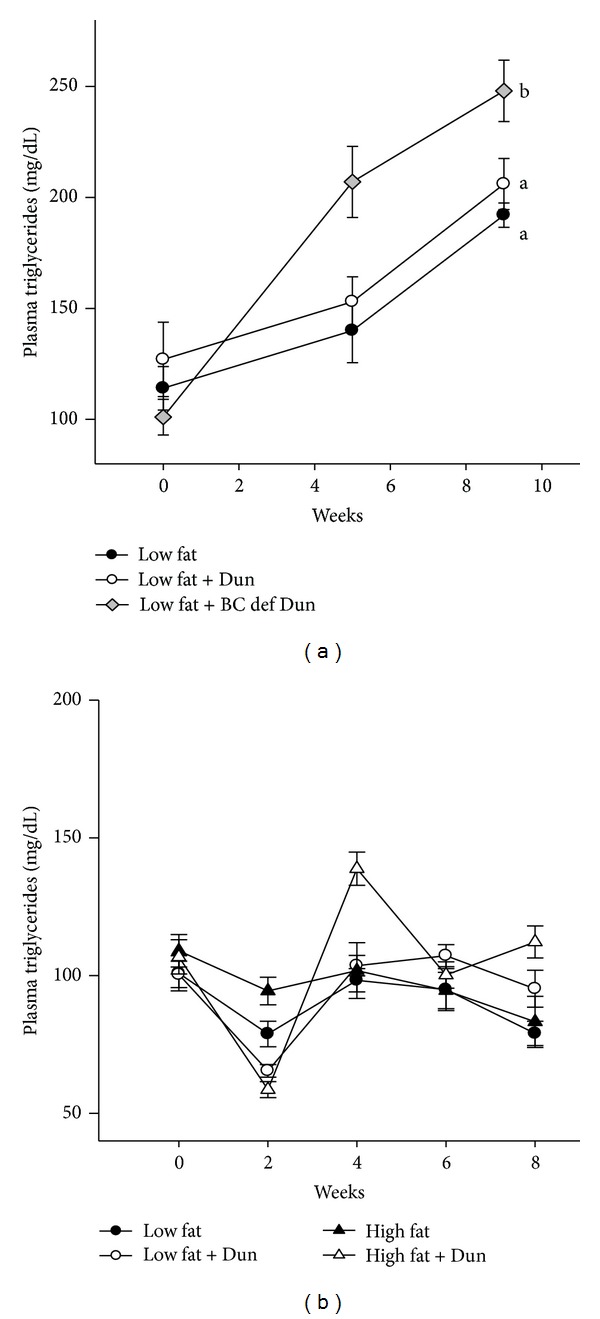
Plasma triglyceride levels in apoE^−/−^ mice fed (a) a low-fat diet, low-fat diet + *Dunaliella* (Dun), and low-fat diet + *β*-Carotene-deficient *Dunaliella* (BC def Dun) (first experiment); (b) low-fat diet, low-fat diet + Dun, high-fat diet or high-fat diet + *Dunaliella* (Dun), (second experiment). Values are means ± SE, *n* = 10–15 in each group. Means at a time without a common letter differ, *P* < 0.05.

To study the effect of *Dunaliella* on plasma lipids and lipoproteins, we measured fasting plasma lipid levels. The *Dunaliella* powder did not affect plasma cholesterol levels in young or old apoE^−/−^ mice fed a low-fat diet (Figures 2(a) and 2(b)). In contrast, in old apoE^−/−^ mice fed a high-fat diet (second experiment), the *Dunaliella* powder significantly inhibited plasma cholesterol elevation compared to control, untreated mice (Figure 2(b)), (598 ± 64 and 942 ± 108 mg/dL, resp., *P* = 0.007). Analysis of plasma lipoproteins showed that the reduction of plasma cholesterol in *Dunaliella*-treated mice is due to reduced cholesterol levels in Very Low Density Lipoproteins (VLDL) (Figure 2(c)). In contrast to the plasma cholesterol lowering effect of *Dunaliella*, there was no effect on plasma triglyceride levels (Figures 3(a) and 3(b)), regardless of the type of diet. Interestingly, a *β*-Carotene-deficient *Dunaliella* powder elevated plasma triglyceride levels (Figure 3(a)).

### 3.3. Atherosclerotic Lesion Area

**Figure 4:**
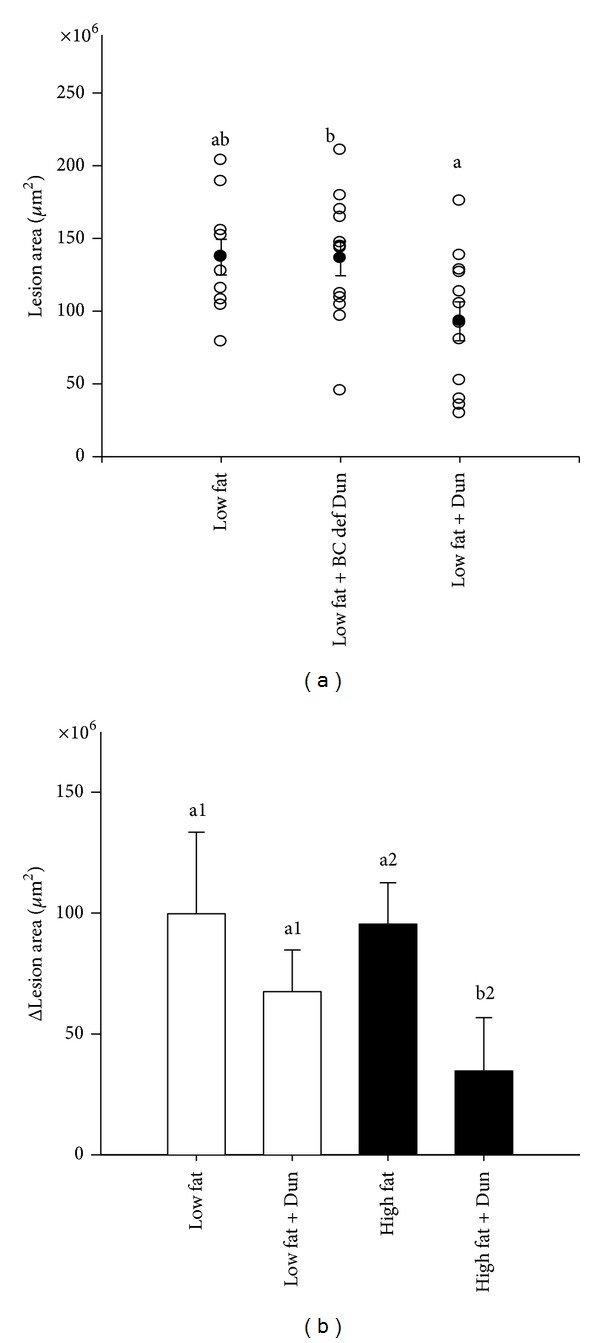
Aortic sinus lesion area in young apoE^−/−^ mice fed (a) a low-fat diet, low-fat diet + *Dunaliella* (Dun), or low-fat diet + *β*-Carotene-deficient *Dunaliella* (BC def Dun) (first experiment), *n* = 10–12 in each group; (b) in old apoE^−/−^ mice fed with a low-fat diet, low-fat diet + *Dunaliella* (Dun), high-fat diet or high-fat diet + *Dunaliella* (Dun) (second experiment), *n* = 15 in each group.

**Figure 5:**
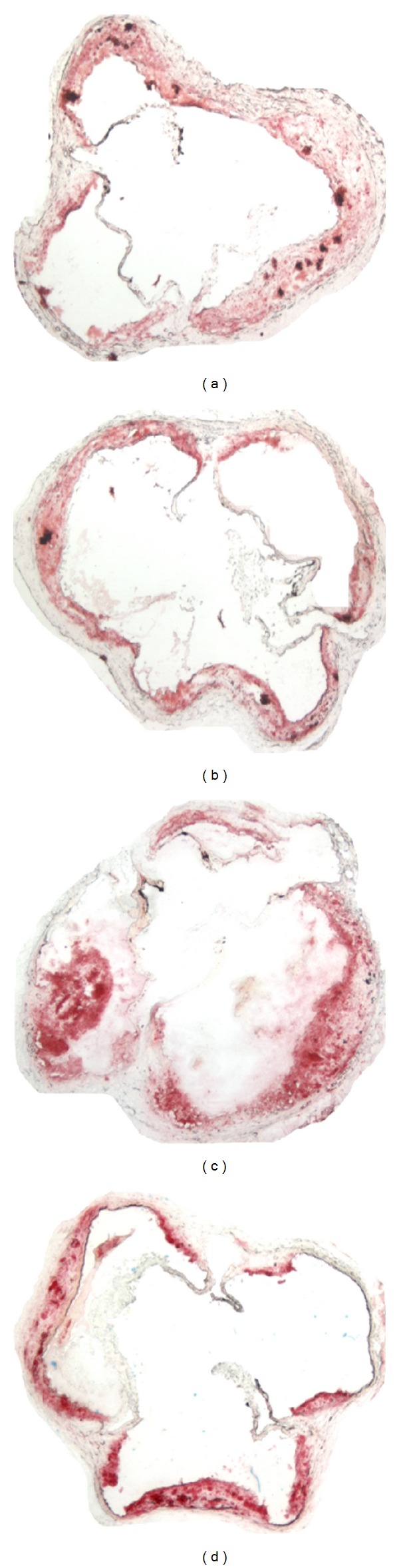
Representative photographs of the aortic sinus lesion in mice fed a low-fat diet (a), low-fat diet + *Dunaliella* (Dun) (b), high-fat diet (c), and high-fat diet + *Dunaliella* (Dun) (d).

In the first experiment, we compared the effect of *Dunaliella* powder containing *β*-Carotene versus *β*-Carotene-deficient *Dunaliella* in young apoE^−/−^ mice. A trend towards a lower lesion area was observed in mice fed low-fat diet, in the *Dunaliella* group compared with the control untreated group (32% reduction, *P* = 0.057). Moreover, aortic sinus lesion area was significantly lower in the *Dunaliella* group compared to the *β*-Carotene-deficient *Dunaliella* group (33% reduction, *P* = 0.045, Figure 4(a)). We further studied the ability of *Dunaliella* to inhibit the progression of established atherosclerosis in old apoE^−/−^ mice. We measured aortic sinus lesion area as a baseline in a subgroup of 7-month-old apoE^−/−^ mice (*n* = 10) and following 8 weeks of treatment in the 4 experimental groups. At the end of the study, we calculated the progression of lesion area in each group compared to the lesion area at baseline. *Dunaliella* significantly inhibited atherosclerosis progression (60% reduction, *P* = 0.038) in mice fed a high-fat diet (Figure 4(b)). The difference between the *Dunaliella* and control groups in mice fed a low-fat diet was not significant (*P* = 0.393) (Figure 5). It should be noted that at this age, the progression of atherosclerosis in the control groups was similar regardless of the type of diet.

## 4. Discussion

The current study demonstrates that a 9-cis-*β*-Carotene-rich diet provided as *Dunaliella* powder lowers plasma cholesterol levels and inhibits atherosclerosis progression in high-fat diet fed apoE^−/−^ mice. It is well known that plasma cholesterol has a major role in atherosclerosis development and that elevated level of plasma LDL cholesterol is the primary target of therapy for the prevention of coronary heart disease. 

In a previous study [14], we showed that *Dunaliella* reduces plasma cholesterol levels and atherosclerotic lesion area in LDL-R^−/−^ mice fed a high-fat diet. In the current work we had two major objectives to study the effect of *Dunaliella* on the progression of established atherosclerosis in the apoE^−/−^ mouse model and to investigate whether the effect of *Dunaliella* is influenced by the dietary fat content. 

 In contrast to LDL-R^−/−^, apoE^−/−^ mice develop atherosclerosis on either low-fat or high-fat diet regimes. In apoE^−/−^ mice fed a low-fat diet a trend towards reduced atherosclerotic lesion area was found between *Dunaliella* and the control, untreated mice. To demonstrate that the *β*-Carotene is the active ingredient in the algal powder, we used additional group of mice, treated with powder exposed to air, in which the *β*-Carotene was totally degraded. We found a significant difference between mice treated with *β*-Carotene-deficient *Dunaliella* and mouse treated with *Dunaliella*. The elevation of plasma triglycerides in mouse treated with *β*-Carotene-deficient *Dunaliella* can be caused by the oxidation products of carotenoids or other ingredients of the alga and highlights the importance of preserving carotenoid preparations against oxidation. The results show that similar to LDL-R^−/−^ mice, *Dunaliella* inhibits atherosclerosis in young apoE^−/−^ mice and that the beneficial effects of the *Dunaliella* powder can be attributed to *β*-Carotene. 

 The effect of *Dunaliella* on atherogenesis was studied in young apoE^−/−^ mice fed low-fat diet. Despite the lack of significant difference in plasma cholesterol levels, there was a trend of reduced lesion area. This reduction can be attributed to the effect of *Dunaliella* on inflammation. We have demonstrated in our previous studies that *Dunaliella* reduced the expression of hepatic and adipose tissue inflammatory genes in mice [14, 16]. The effect of *Dunaliella* on prevention of progression of atherosclerosis was studied in old apoE^−/−^ mice with established atherosclerotic lesions fed a low- or high-fat diet. In apoE^−/−^ high-fat diet-treated mice, *Dunaliella* significantly reduced atherosclerotic lesion area, to the same extent as in LDL-R^−/−^ mice. Importantly, *Dunaliella* did not affect the plasma cholesterol levels in mice consuming a low-fat diet but did reduce plasma cholesterol in high-fat-fed mice. In a former study, we demonstrated that *Dunaliella* does not affect cholesterol absorption from the diet [14]. Further studies are needed to elucidate in what manner *Dunaliella* reduces plasma cholesterol levels in mice fed a high-fat diet. Our results are in accordance with recent studies showing that carotenoids and their metabolites influence lipid metabolism in animal models [17–19].

 In conclusion, our results suggest that in contrast to previous studies, showing the failure of synthetic all-trans-*β*-Carotene to inhibit atherosclerosis in animal models [12, 14] and a lack of benefit on cardiovascular disease [3, 4], a diet containing natural carotenoids has the potential to inhibit atherosclerosis progression, particularly in high-fat diet regime.
